# Common Handling Procedures Conducted in Preclinical Safety Studies Result in Minimal Hepatic Gene Expression Changes in Sprague-Dawley Rats

**DOI:** 10.1371/journal.pone.0088750

**Published:** 2014-02-14

**Authors:** Yudong D. He, Christine M. Karbowski, Jon Werner, Nancy Everds, Chris Di Palma, Yuan Chen, Marnie Higgins-Garn, Sandra Tran, Cynthia A. Afshari, Hisham K. Hamadeh

**Affiliations:** 1 Comparative Biology and Safety Sciences, Amgen Inc., Seattle, Washington, United States of America; 2 Comparative Biology and Safety Sciences, Amgen Inc., Thousand Oaks, California, United States of America; Indian Institute of Toxicology Reserach, India

## Abstract

Gene expression profiling is a tool to gain mechanistic understanding of adverse effects in response to compound exposure. However, little is known about how the common handling procedures of experimental animals during a preclinical study alter baseline gene expression. We report gene expression changes in the livers of female Sprague-Dawley rats following common handling procedures. Baseline gene expression changes identified in this study provide insight on how these changes may affect interpretation of gene expression profiles following compound exposure. Rats were divided into three groups. One group was not subjected to handling procedures and served as controls for both handled groups. Animals in the other two groups were weighed, subjected to restraint in Broome restrainers, and administered water via oral gavage daily for 1 or 4 days with tail vein blood collections at 1, 2, 4, and 8 hours postdose on days 1 and 4. Significantly altered genes were identified in livers of animals following 1 or 4 days of handling when compared to the unhandled animals. Gene changes in animals handled for 4 days were similar to those handled for 1 day, suggesting a lack of habituation. The altered genes were primarily immune function related genes. These findings, along with a correlating increase in corticosterone levels suggest that common handling procedures may cause a minor immune system perturbance.

## Introduction

**Figure 1:**
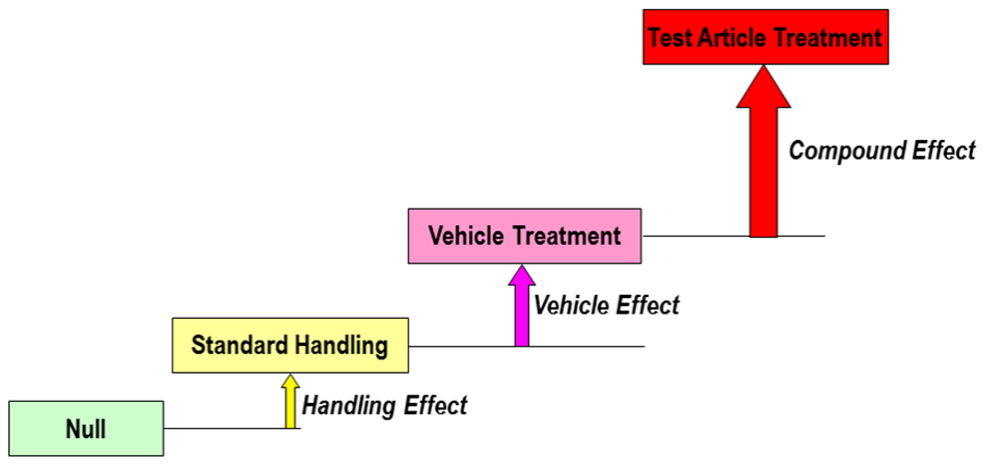
An additive model for the gene expression changes detected in toxicology studies. Gene changes detected in toxicology studies are a combination of effects resulting from handling procedures as well as treatment with compound and vehicle.

Molecular endpoints are frequently monitored in tissues derived from *in vivo* studies. Incorporation of global gene expression analysis in rodent *in vivo* studies has become common practice in safety assessment and other toxicology fields with the goal of predicting potential compound associated liabilities and understanding molecular mechanisms underlying observed toxicities [1]–[3]. Numerous studies have reported gene expression changes in rat livers in response to various compound treatments [1]. During drug development in particular, the liver has been a major focus of toxicogenomics efforts with the goal of reducing hepatotoxicity which is a common cause for drug failure. These results are potentially affected by common handling procedures, including compound administration, restraint, and blood collection. Gene expression changes detected in toxicology studies can be viewed as a composition of effects resulting from handling of animals as well as treatment with compound and vehicle (Figure 1). While studies have documented the effects of handling on blood pressure, heart rate, and glucocorticoid concentrations in laboratory animals [4]–[6], no work has been reported thus far that has systematically examined the effects of common handling procedures on hepatic gene expression in rats.

This study was conducted in order to understand the gene expression changes detected in a typical short duration preclinical study by interrogating the gene changes in rat liver associated with common handling procedures. Female Sprague-Dawley rats were subjected to common handling procedures for 1 or 4 days in order to mimic a standard 4 day multiple dose study, in addition a group of control animals that were not subjected to handling procedures. Genome-wide gene expression data were generated from liver tissues, and significantly altered genes were statistically identified and studied for their membership in biological pathways. The perturbations in pathways due to handling procedures were minor in magnitude and consistent with minor differences in clinical pathology or histopathology measurements between the handled and non-handled groups. Pathway analysis of altered genes revealed that there were minor decreases in complement genes and increases in a small number of additional immune function related genes which may indicate a minor immune perturbance in the liver. This molecular portrait of gene expression perturbations describes gene expression changes associated with common handling procedures, which will be helpful for the biological interpretation of the overall response to compound treatment in toxicology studies.

## Materials and Methods

### Ethics Statement

All research protocols were approved by the Amgen, Inc. (Seattle, WA) Institutional Animal Care and Use Committee.

### In Vivo Study

Female Sprague Dawley rats (rattus norvegicus; Charles River Laboratories, Hollister, CA), approximately 10 weeks of age, were cared for as described in the *Guide for the Care and Use of Laboratory Animals, 7^th^* Edition [7]. Animals were group housed at an AAALAC, Intl- accredited facility in non-sterile, ventilated, micro-isolator cages on corn cob bedding.

Animals had *ad libitum* access to pelleted feed (2020X Teklad; Harlan Laboratories Inc., Madison, Wisconsin) and water (reverse osmosis-purified) via an automatic watering system. Animals were maintained on a 12∶12 hour light: dark cycle in rooms at 71 degrees C +/−5 degrees and 40% humidity (range 30–70%) and had access to enrichment opportunities (enrichment tubes and nylabones). All animals were determined specific pathogen free.

Rats were divided into three groups (n = 10 per group) based on body weights (Table 1). One group was not subjected to handling procedures and served as controls. Animals in the other two groups were weighed and restrained with Broome restrainers which consisted of removing the animal from their home cage, recording body weight, restraining for dose administration, and then returning the animal to its home cage. Dose administration consisted of water via oral gavage daily for 1 day or 4 days with tail vein blood collections at 1, 2, 4, and 8 hours postdose on days 1 or 4. Animals were euthanized by exsanguination under deep isoflurane anesthesia on day 2 or 5, and blood samples were collected for clinical pathology evaluation. Gene expression profiles, traditional clinical pathology endpoints, and serum corticosterone concentrations were measured. Adrenals and liver were evaluated microscopically.

**Table 1:** Study design.

Group	# Rats	Scheduled Euthanasia	Assessment of Body Weight(s)	Restraint and Gavage^a^	Multiple IV Blood Collections^b^
1	10	Day 2	No	No	No
2	10	Day 2	Yes	Yes	Yes
3	10	Day 5	Yes	Yes	Yes

a Restraint procedures consisted of removing the animal from their home cage, recording body weight, restraining for dose administration and then returning the animal to its home cage. Animals receiving gavage were administered water via oral gavage at a dose volume of 10 mL/kg.

b Restraint in Broome restrainers followed by 0.16 mL blood collected from the lateral tail vein into serum separator tubes at 1, 2, 4, 8 hours post dose on days 1 and 4 for Groups 2 and 3, respectively.

### RNA Isolation

Total RNA was isolated from ∼30 mg pieces of the left liver lobe according to the RNeasy extraction procedure (Qiagen, Valencia, CA). Snap frozen tissues were transferred to Lysing Matrix D tubes containing RLT lysis buffer supplemented with beta-mercaptoethanol and homogenization was performed using the FastPrep® instrument (MP Biomedicals, Solon, OH). An on-column DNase digestion was performed to remove any residual genomic DNA contamination. RNA concentration and yield were measured and quality of the nucleic acid samples was evaluated with the Agilent Bioanalyzer (Agilent Technologies, Santa Clara, CA). All samples were of good quality, as evidenced by distinct ribosomal 18S and 28S peaks, low baseline and high RIN values.

### Gene Expression Data Generation

Microarray profiling was outsourced to Expression Analysis (Durham, NC). Biotinylated cRNA was generated from 100 ng of total RNA using the GeneChip 3′ IVT Express Target Labeling method (Affymetrix, Santa Clara, CA). For each sample, 10 ug of biotinylated cRNA spiked with hybridization controls (bioB, bioC, bioD and cre) was hybridized to an Affymetrix GeneChip® Rat Genome 230 2.0 array for 16–18 hours at 45°C. Following hybridization, arrays were washed and stained in a GeneChip® Fluidics Station and scanned with a GeneChip® Scanner 3000 (Affymetrix). Quality checks and data analyses were carried out using Affymetrix GeneChip Operating Software (GCOS).

### Outlier/Abnormalities Identification

The quality control parameters provided by Expression Analysis were inspected for outliers or patterns. In addition, all samples were analyzed using principal component analysis (PCA) to identify samples which clearly do not group with the other samples in the space defined by the first three principal components. Three samples showed genome-wide profiles that slightly differed from the rest of the seven samples in the same control group. However, we found no sample-QC metrics based evident to support excluding these samples as outlier samples. Consequently, all 30 samples in the study were kept in the subsequent data analysis.

### Gene Expression Data Preprocessing

A Log_10_(Ratio) was computed for each gene using Rosetta Resolvers’ (version 7.2, Rosetta Biosoftware, Seattle, WA*)* error-weighted ratio builder between each individual profile and the mean of the control group. A Log_10_(Ratio) was also computed for each individual control profile versus the mean of the control group for variability assessment. The output of the ratio forming is an estimate of the Log_10_(Ratio) for each gene between each profile and the mean of the control group and a p-value indicating the confidence that the Log_10_(Ratio) is different than 0 (that is the ratio is different than 1). Subsequent statistical analyses were based on values of Log_10_(Ratio) of each genes in each rat liver sample.

### Identification of Signature Genes

In order to identify genes with differences in expression levels among the three groups (control, 2-day handling, and 5-day handling), the Log10(Ratio) data were analyzed by a 3-group one-way ANOVA. Three possible pair-wise comparisons were also performed separately using a t-test for differences between Group 1 and Group 2 (stress response at Day 1), between Group 1 and Group 3 (stress response at Day 4), and between Group 2 and Group 3 (time dependence of stress response from Day 1 to Day 4). A non-parametric analysis was also used to capture systematic changes between all three pair-wise comparisons. Developed in-house, this method was proven to be more robust for capturing the group differences against potential outliers within groups [8]. No minimum fold change was required in the gene selection and the corresponding false discovery rates (FDR) were also estimated for each comparison at our chosen p-value cutoff in Table 2. The resulting 441 genes from the union of ANOVA results with p-value <0.001 and non-parametric analysis of pair-wise comparisons with p-value <0.005 were used for the pathway analysis. For full data please see GEO (GSE46702).

**Table 2:** Clinical and anatomic pathology results.

Treatment:		No Handling	Handling Procedures	Handling Procedures
Necropsy Day:		2	2	5
Hematology				
	RBC x106/µL	7.27	6.85*	6.99*
	HGB g/dL	13.8	12.6*	13.0*
	HCT %	40.6	37.5*	38.3*
	PLT x103/µL	1027	1163*	1153
Clinical Chemistry				
	ALT U/L	39	37	31*
	GLDH U/L	6.8	10.4*	10.7*
Corticosterone				
	1 hour post last dose	NA	250	195
	2 hours post last dose	NA	180	102
	4 hours post last dose	NA	179	232
	8 hours post last dose	NA	720	513
	24 hours post last dose	609	608	547
Organ Weights a No. evaluated:		7	10	10
	Adrenal	0.063	+13%*	+12%
	Adrenal TBW%	0.029	+13%*	+14%
Histopathology ^b^ No. evaluated:		7	10	10
	Adrenal			
	Hypertrophy, cortex			
	Minimal	0	3	3

a For controls, group mean is shown; for groups subjected to handling procedures, percent differences from controls are shown. ^b^ Incidence. NA = Not available. * p<0.05 by Two-tailed Student’s t-test when compared to control group. Numbers underlined are considered related to handling procedures.

### Estimate of False Discovery Rate

**Figure 2:**
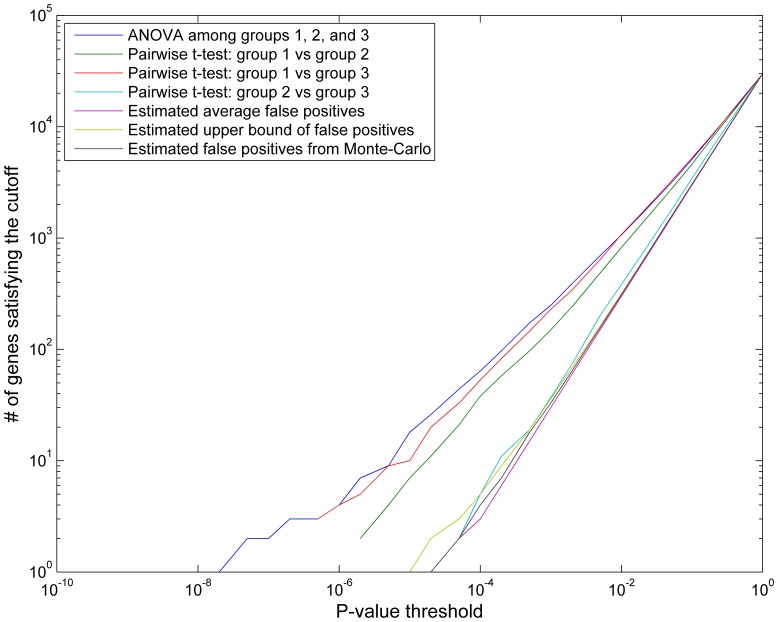
Number of signature genes as a function of p-value cutoff for various statistical tests. More signature genes would be reported with higher FDR by applying a less stringent cutoff on p-value. We use p-value cutoff at 0.001 with FDR ∼12%. Note the legend on the upper-right corner explains the meaning of each line.

For a specified p-value cutoff (*P_th_*), a number of genes (*N_obs_*) were obtained as significant genes. In Figure 2, *N_obs_* is plotted in the y-axis as a function *P_th_* in the x-axis for the results from 3-group one-way ANOVA and three pair-wise t-test comparisons. For each case, an upper bound can be placed on the false discovery rate (*FDR*) by estimating the type I error associated with each observation. Three methods were used to estimate the FDR. First, at each *P_th_*, the number of false positives was estimated on average to be *N_mean_* =  N_total_*P_th_ in which *N_total_* is the total number of genes in the selection pool (∼30,000). Second, an upper bound of false positives can also be determined as *N_upper_ = N_mean_+sqrt(N_mean_)*. Thirdly, false positives, *N_mc_*, can be estimated from Monte-Carlo simulation of random permutations between two groups. The *FDR* can then simply be approximated as *FDR =  N_mean_/N_obs_, N_upper_/N_obs_, N_mc_/N_obs_* at each *P_th_*. This means that for criterion with *P_th_* at 0.001, up to 30 genes (or 12%) of the identified 249 significant genes are potentially false discoveries. A more stringent cutoff would lead to fewer false discoveries but also reduce the number of signature genes to investigate. Conversely, a less stringent cutoff would lead to more signature genes with a greater number of false discoveries. Therefore, the cutoff p<0.001 with FDR = 12% provides a more optimal balance between false discoveries and signature genes.

### Pathway Analysis

Enriched gene sets associated with each of the two signature sets were identified using Fisher’s exact test as well as the Random Set method [9]. Molecular pathways as defined in the Ingenuity Pathways Analysis database version 8.5 (Ingenuity Systems, Redwood City, CA) were used. Top-ranked pathways were subject to further investigation using supporting evidence from the literature. Reported gene symbols are human orthologs. Note that only approximately 50% of sequences that are measured in this experiment are mapped to annotated pathways in IPA. The remaining sequences are either not annotated or not members of IPA pathways.

## Results

### In Vivo Study

The study design consisted of 3 groups, each with 10 female Sprague-Dawley rats (Table 1). The objective was to evaluate differences in gene expression among the three groups (1-day handling, 4-day handling, and 1-day without handling). Ten (10) animals in each group were used to gain enough power to detect small changes in gene expression between these groups given the potential subtlety of the effect. Clinical pathology changes were minimal and limited to decreased RBC mass parameters, increased platelet counts, and increased glutamate dehydrogenase activity in animals handled for 1 or 4 days; alanine aminotransferase was minimally decreased in animals handled for 4 days only (Table 2). Corticosterone concentrations, measured in handled animals only, were increased compared to baseline at 1 and 2 hours after dosing on day 1 but not on day 4. There was minimal hypertrophy of the adrenal cortex in 3 animals from each group of handled animals that was not observed in the unhandled animals.

### Identification of Differentially Expressed Genes

**Figure 3:**
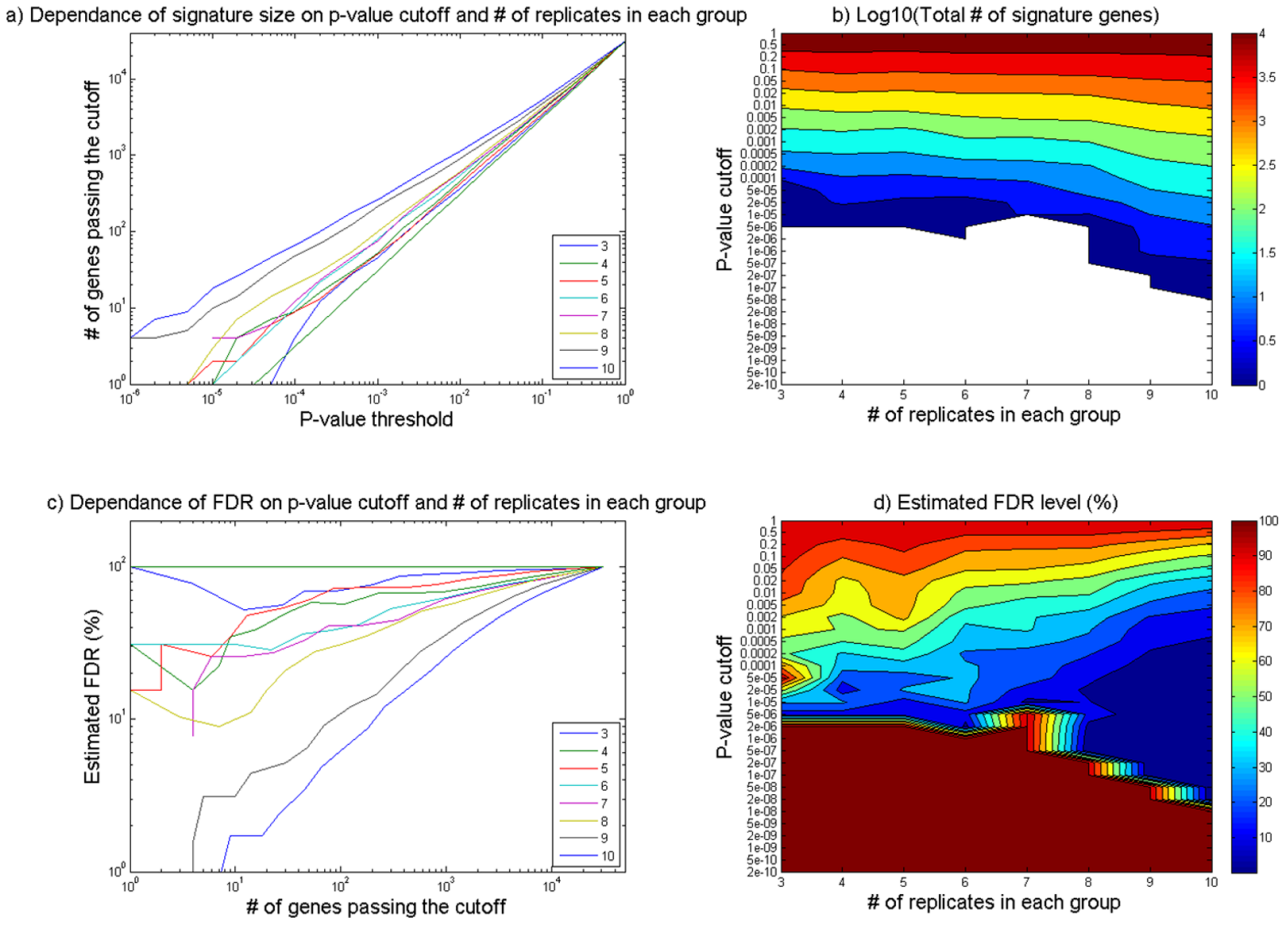
Dependence of signature size and FDR on p-value cutoff and number of replicates. Each group is shown separately (a and b) and simultaneously (c and d).

Three statistical methods were used to identify genes that illustrate differences in their expression among experimental groups. All methods are viewed complementary to each other. Monte-Carlo methods were used to explore the dependency of signature size (N_sig_) and false discovery rate (FDR) on the p-value cutoff (P_th_) and number of replicates (*n*) in each group for *n* ranging from 3 to 10. For a given choice of *n* (ranging from 3 to 10), increasing selection stringency (decreasing P_th_) led to decreased N_sig_, as illustrated in Figure 3 (a). For the same p-value cutoff, in the case illustrated with *n* ranging from 3 to 10, we obtained a larger number of significant genes with a greater *n*. The heatmap in Figure 3 (b) illustrates the dependency of N_sig_ on both *n* and P_th_ in a two-dimensional plot. Figure 3 (c) and (d), also shows the dependency of FDR associated with each N_sig_ on *n* and P_th_ in one two dimensional plots. Together, these figures provide a data-based performance estimate for the choice of number of animals per group (*n*) in the experimental design as well as the choice of a p-value cutoff. They indicate that a sample size of 10 replicates per group results in identification of a higher number of genes while the p-value choice of 0.001 results in identification of genes with a low number of false positives.

**Figure 4:**
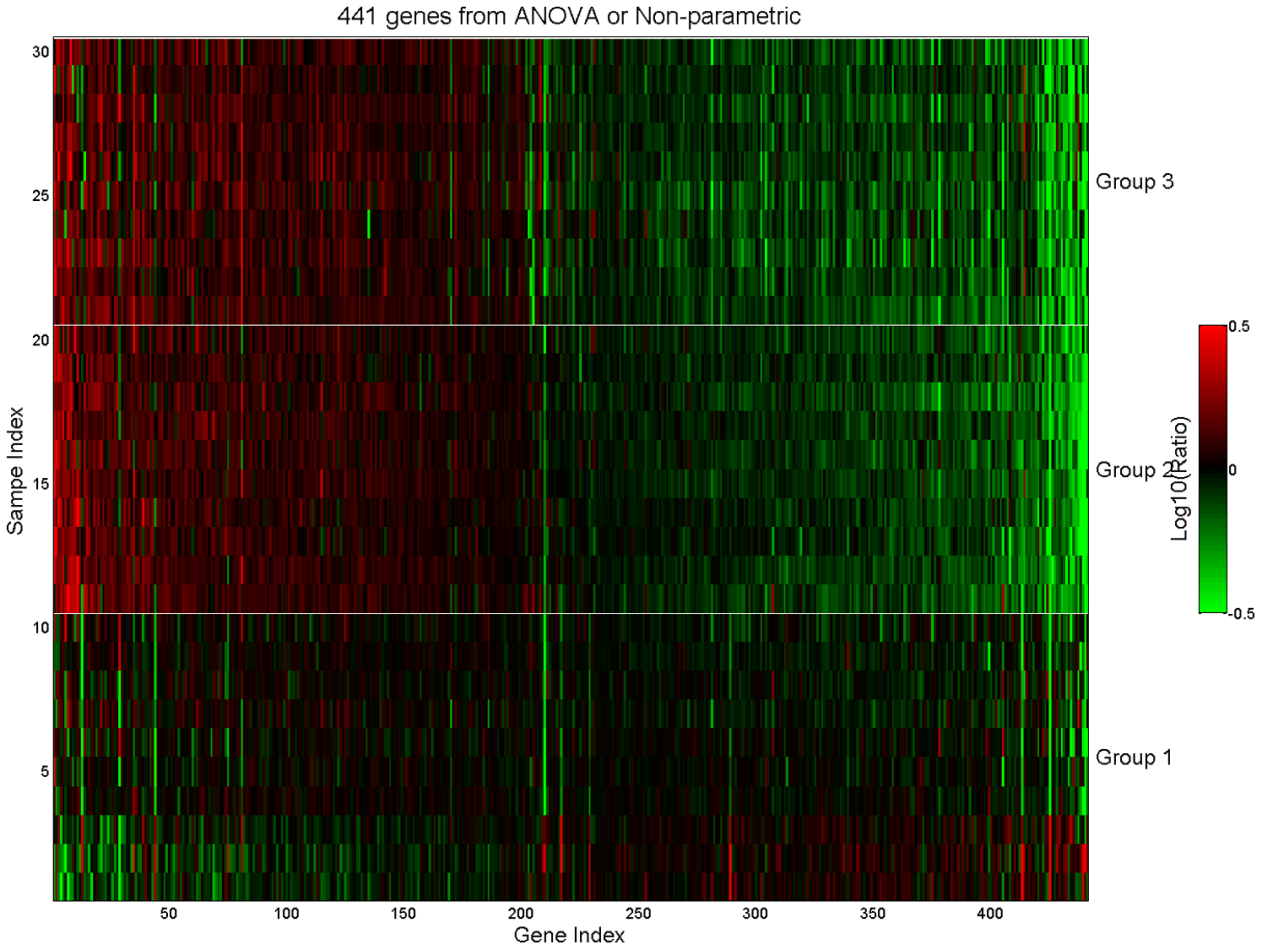
Heatmap of signature genes used in pathway analysis. The 441 signature genes were selected based on the union of ANOVA and non-parametric analyses for pathway analysis. Data is expressed as Log10(Ratio) data. Genes were sorted based on their significance from corresponding statistical tests from left to right for up-regulated and down-regulated genes, respectively. Groups 1–3 are defined in Table 1 with *n* = 10 in each group.

To detect all genes of interest, our analysis focused on the union of genes identified by the ANOVA and non-parametric analysis of pair-wise comparisons at a p-value cutoff of 0.001. Less than 1% of genes were significantly altered upon comparison of any of the experimental conditions. A slightly greater percentage of genes with a more reliable false discovery rate (FDR) were significantly altered in animals that were handled when compared to unhandled animals (group 1 versus group 2 or 3) than between the two groups of animals that were handled (group 2 versus 3; Table 3). Comparison of the 441 significantly altered genes resulting from the union of statistical methods highlights that a majority of genes were altered similarly increased or decreased between animals handled for 2 or 5 days when compared to the unhandled controls (Figure 4).

**Table 3:** Percentage of differentially expressed genes for each comparison.

Comparison	% of genes significant with p<0.001	Estimated FDR
Group 2 vs Group 1	0.83%	12.00%
Group 3 vs Group 1	0.96%	10.42%
Group 3 vs Group 2	0.10%	85.71%

### Key Findings from Significantly Altered Pathways

Differentially expressed sequences were analyzed by Ingenuity Pathway Analysis (IPA) to enrich for pathways that were affected in animals that underwent common handling procedures for either 1 or 4 days when compared to unhandled animals. The resulting pathways were used as a guide to group altered genes perturbed by the common handling procedure into general biological processes.

Overall, gene changes were relatively small in magnitude with fold changes for described genes ranging from −1.1 to 1.8 fold (Table 4). The top scoring pathway result from IPA was the complement pathway which was comprised primarily of genes that were significantly decreased. The complement components C5, C9, complement factor B (*CFB*), and complement component 4 binding protein alpha (*C4BPA*) were all decreased while the C1q, C-chain gene (*C1QC*) was increased. C1QC is involved in activation of the classical pathway of the complement system and increased expression may represent compensation for the decreased expression of other complement pathway genes. Additional immune system related genes were significantly altered and primarily increased such as IL-1 alpha (*IL1A*), the chemokine receptor *CCRL1*, *CD7*, and the major histocompatibility I complex gene *HLA-Cc*, in addition to the leukocyte extravasation related genes junctional adhesion molecule (*JAM2*) and intracellular adhesion molecule 1 (*ICAM1*). Interestingly, the chemokine *CXCL12* was decreased. Collectively, these gene changes indicate a minor immune system perturbation following handling of animals for 2 or 5 days.

**Table 4:** Highlighted genes that were differentially expressed in animals subjected to common handling procedures when compared to unhandled controls.

Biological Process	Gene Symbol	Gene Name	Fold Change
Immune Function	*CD7*	Cd7	+1.2–1.5
Immune Function	*CCRL1*	Chemokine (C-C motif) receptor-like 1	+1.2–1.7
Immune Function	*HLA-C*	Major histocompatibility complex, class I, C	+1.2–1.4
Immune Function	*ICAM1*	Intracellular adhesion molecule 1	+1.2–1.3
Immune Function	*IL1A*	Interleukin 1, alpha	+1.2–1.8
Immune Function	*JAM2*	Junctional adhesion molecule 2	+1.2–1.3
Immune Function/Complement System	*C4BPA*	Complement component 4 binding protein	−1.2
Immune Function/Complement System	*C5*	Complement component 5	−1.3
Immune Function/Complement System	*C9*	Complement Component 9	−1.1–1.2
Immune Function/Complement System	*CFB*	Complement factor B	−1.2–1.2
Immune Function/Complement System	*CXCL12*	Chemokine (C-X-C motif) ligand 12	−1.1–1.2
Apoptosis & Cell Cycle	*BAX*	Bcl2-associated X protein	+1.3
Apoptosis & Cell Cycle	*CAPNS1*	Calpain, small subunit 1	+1.1–1.4
Apoptosis & Cell Cycle	*CCND3*	Cyclin D3	+1.1–1.3
Apoptosis & Cell Cycle	*CCNG1*	Cyclin G1	+1.2–1.3
Apoptosis & Cell Cycle	*CUL1*	Cullin 1	−1.1
Apoptosis & Cell Cycle	*HTRA2*	HtrA serine peptidase 2	+1.1
Apoptosis & Cell Cycle	*PCNA*	Proliferating cell nuclear antigen	+1.1–1.2
Apoptosis & Cell Cycle	*PERP*	TP53 apoptosis effector	+1.1–1.2
Apoptosis & Cell Cycle	*TGFBR1*	Transforming growth factor beta receptor 1	−1.1–1.2
Protein Ubiquitin Pathway/Apoptosis & Cell Cycle	*BAG1*	Bcl2-associated athanogene	+1.1
Protein Ubiquitin Pathway	*Psmd9*	Proteasome 26S subunit, non ATPase, 9	+1.3
Protein Ubiquitin Pathway	*UBE2V1*	Ubiquitin-conjugating enzyme E2 variant 1	+1.2–1.4
Protein Ubiquitin Pathway	*UCHL3*	Ubiquitin carboxyl-terminal esterase L3	+1.2
Oxidative Stress	*HMOX1*	Heme oxygenase 1	+1.1
Oxidative Stress	*GSS*	Glutathione synthetase	+1.3–1.4
Oxidative Stress	*GSTM4*	Glutathione S-transferase mu 4	+1.3–1.4
Oxidative Stress	*GSTP1*	Glutathione S-transferase pi 1	+1.1–1.2

Additional gene expression changes were noted in a small number of genes involved in apoptosis or cell cycle. Increased expression was noted in the apoptosis related genes Bcl2 associated X protein (*BAX*), HtrA serine peptidase 2 (*HTRA2*), *PERP*, and calpain 1 (*CAPNS1*). Increased expression was also noted in the proliferating cell nuclear antigen (*PCNA*), which is involved in DNA synthesis, and cyclin G1 (*CCNG1*), which may play a role in G2/M growth arrest, while the transforming growth factor beta receptor 1 (*TGFBR1*) was decreased. Alterations were noted in a minor number of cell cycle progression genes with increased expression of cyclin D3 (*CCND3*), which is involved in the G1/S transition, and decreased expression of its inhibitor *CUL1*.

In addition to apoptosis and cell cycle gene changes, there was increased expression in a small number genes related to the ubiquitin pathway; the proteasome 26S subunit *PSMD9*, the ubiquitin conjugating enzyme *UBE2V1*, and the ubiquitin carboxyl-terminal esterase gene *UCHL3*. There was also increased expression of the Bcl2 associated athanogene *BAG1* which has an anti-apoptotic role in addition to mediating ubiquitin conjugation of unfolded proteins. Additional increases were observed in a small number of genes involved in oxidative stress such as glutathione synthetase (*GSS*), glutathione transferase genes (*GSTM4*; *GSTP1*), and heme oxygenase (*HMOX1*). These gene changes may indicate minor cell cycle alterations along with increased protein turnover and a minor oxidative stress response. However, these changes were minor in magnitude and there were no correlating microscopic changes observed in the liver.

## Discussion

This study was designed to investigate the baseline gene expression changes in livers of rats subjected to common handling procedures in a standard short term multiple dose level preclinical study. The strengths of this study include the experimental design and choice of replicates in each group in addition to the chosen p-value cutoff for identification of significantly altered genes. An *n* of 10 was chosen for each treatment group in order to have power to detect subtle gene expression changes. Monte-Carlo methods exploring the dependency of signature size (N_sig_) and false discovery rate (FDR) on the p-value cutoff (P_th_) and number of replicates (*n*) in each group were employed to determine the validity of this choice. These data-driven analyses substantiate the choice of 10 replicates per group in order to identify a larger number of gene changes at a given p-value cutoff. They also illustrate the practical trade-off between N_sig_ and FDR for a given choice of *n*. For example, one can always obtain more signature genes (larger N_sig_) by placing a less stringent cutoff (greater P_th_). However, by doing so, one should also expect a higher percentage of spurious signature genes as represented by a higher FDR. In this study a p-value cutoff of 0.001 was chosen in order to reduce the number of spurious signature genes retrieved, thus increasing the confidence that these signature genes represent true biological effects.

Relatively few significant gene expression changes were identified in the livers of animals that underwent common handling procedures for either 1 or 4 days when compared to unhandled animals at day 2 (∼1%). However, by taking the union of two complementary statistical methods at an FDR of approximately 10%, these ∼400 genes likely represent true biological changes with a low number of false positives. Conversely, comparison of the animals handled for 2 days with those handled for 5 days resulted in identification of only approximately 0.1% of genes demonstrating significantly differential expression which are more likely to represent false positives as indicated by a higher FDR. Changes were similar in animals handled for 1 day and those handled for 4 days, implying a lack of sensitization or habituation to handling in a short time-frame. Of further interest would be whether this response would subside upon a much longer period of common handling procedures.

Pathway analysis demonstrated that handling procedures led to altered expression of immune function related genes with a general decrease in multiple complement system genes as well as a small number of additional immune function related genes. Collectively, these changes likely indicate a minor immune perturbation in the liver following handling procedures. The effects of handling laboratory animals and the resulting alterations in immune response has been documented previously [4]. In general, mild and or acute stressors enhance humoral immune responses while severe or long-term stressors can be immunosuppressive [10]–[18]. Stress-related changes in the functional capacity of the immune system have been discussed in a recent review article [19] and include decreased immune cell numbers and functions, decreased response to vaccinations, and increased immunosuppressive pathways and proinflammatory cytokines. Effects of chronic stress on immune function have been well-documented in humans and include effects on both the innate and adaptive immune system [20]. As summarized by Gouin, specific findings include attenuated responses to vaccination, poorer wound healing, exaggerated release of inflammatory mediators, and premature aging of the immune system. Endogenous glucocorticoids are essential for antibody responses and adrenalectomy or anti-glucocorticoid receptor treatment decreases IgM and IgG responses which can be restored by corticosterone replacement [21]. Activation of the complement cascade occurs rapidly [22] and is reported following acute psychological stress in humans with increased circulating levels of C3a, C5a, and Factor Bb [23]. Thus, perturbation of immune system related genes in the liver and a post-handling increase in corticosterone levels at day 2 are consistent with previous reports of acute stress-related effects. Liver gene expression changes were measured in necropsied livers on the day following dose administration and tail vein blood collections. Therefore, the decreased expression of complement genes in light of an increase in other immune genes may reflect a transcriptional feedback loop.

In conclusion, the results reported here highlight gene expression changes that occur in laboratory animals during routine handling procedures. These gene expression changes can be viewed as baseline changes upon which additional transcript level alterations resulting from vehicle and/or compound related effects are layered. One can envision the composition of these effects as depicted in Figure 1. This simple additive model illustrates how transcriptional effects at various levels on preclinical animals may add up. If control animals in a study are subjected to the same handling procedures these gene expression changes may be normalized as vehicle related changes are normalized. However, more complex models and interactions with vehicle and/or compound related gene changes cannot be excluded and may warrant further investigation. Synergistic or subtractive interactions with compound mediated effects may confound interpretation of gene changes in a study. We propose to monitor these 441 “rat stress related genes” in historical and future experiments to better understand the effects of these genes on interpretation of compound induced gene expression changes.
